# Diagnostic value and cost-benefit analysis of 24 hours ambulatory blood pressure monitoring in primary care in Portugal

**DOI:** 10.1186/1471-2261-13-57

**Published:** 2013-08-12

**Authors:** Paulo Pessanha, Manuel Viana, Paula Ferreira, Susana Bertoquini, Jorge Polónia

**Affiliations:** 1 Health Center of São João, Porto, Portugal; 2 Faculdade Psicologia e Ciências da Educação, Bolseira FCT, Porto, Portugal; 3 Faculdade de Medicina da Universidade do Porto, Porto, Portugal

**Keywords:** Hypertension, Ambulatory blood pressure monitoring (ABPM), General practice/Family medicine, White coat hypertension (WCH), Cardiovascular risk factors, ABPM cost-benefit

## Abstract

**Background:**

Hypertensive patients (HTs) are usually attended in primary care (PC). We aimed to assess the diagnostic accuracy and cost-benefit ratio of 24-hour ambulatory blood pressure monitoring (ABPM) in all newly diagnosed hypertensive patients (HTs) attended in PC.

**Methods:**

In a cross-sectional study ABPM was recorded in all 336 never treated HTs (Office BP ≥140 and/or ≥ 90 mm Hg) that were admitted during 16 months. Since benefits from drug treatment in white-coat hypertension (WCH) remain unproven, a cost benefit estimation of a general use of ABPM (vs absence of ABPM) in HTs was calculated comparing the cost of usual medical assistance of HTs only diagnosed in office with that based both on refraining from drug treatment all subjects identified as WCH and on the reduction by half of the frequency of biochemical exams and doctor visits.

**Results:**

Women were 56%, age 51 ± 14 years and BMI 27 ± 4 Kg/m2. Out of these, 206 were considered as true HTs, daytime ABPM ≥ 135 and/or ≥85 mm Hg and 130 (38,7%) were identified as having white coat hypertension (WCH), daytime ABPM <135/85 mm Hg. Versus HTs, WCH group showed higher percentage of women (68% vs 51%) and lower values of an index composed by the association of cardiovascular risk factors. We estimated that with ABPM total medical expenses can be reduced by 23% (157.500 euros) with a strategy based on ABPM for 1000 patients followed for 2 years.

**Conclusions:**

In PC, the widespread use of ABPM in newly diagnosed HTs increases diagnostic accuracy of hypertension, improves cardiovascular risk stratification, reduces health expenses showing a highly favourable benefit-cost ratio vs a strategy without ABPM.

## Background

Hypertension (HT) is a recognized public health problem and is considered the leading cause of death throughout the world [1,2]. It is particularly responsible for the occurrence of fatal and non fatal cardiovascular events and deaths, including stroke and coronary heart disease [1,2]. HT is a chronic disease with an estimated prevalence of 40-45% in adults [3-5] that in most cases requires periodic monitoring, non-pharmacological and pharmacological therapy for a lifetime [1-5]. The diagnosis of hypertension is associated with personal, social and economic disturbances with a noteworthy impact to the patient, to the health system and to the society [1-5]. General Practionerrs (GP) have an important role in the global approach to the hypertensive patient, concerning the diagnosis, treatment and regular monitoring. Actually, the great majority of the hypertensive situations are diagnosed and followed by the GP in primary care. It is now generally recognized that blood pressure (BP) measurement in office has several limitations. In recent years it has been shown that the evaluation of BP with ABPM for 24 hours has several advantages as compared to the BP measurement in office, namely a greater validity and reproducibility and higher predictive value of fatal and non-fatal cardiovascular events [4,6-15]. Furthermore, ABPM allows multiple BP measurements during 24 h that may be more representative of the daily BP burden, permits the evaluation of BP during the daily activities away of the BP alert reaction observed in office and allows the assessment of BP during sleep [9,11,14,16]. Also, ABPM may identify some BP profiles such as the nocturnal fall of BP and the morning rise of BP, which abnormal patterns have been shown to be independent predictors of target-organ damage and of risk of cardiovascular events [17-20]. Besides, ABPM allows the identification of individuals with the so called White Coat Hypertension (WCH) who shows hypertensive values in office but normal BP outside the doctor’s office [5,10,21,22]. In the absence of target organ damage, WCH show an estimated prevalence between 18 and 40% [3,5] is associated with a low cardiovascular risk [1,14,23-25] for which evidence of benefit from drug treatment is lacking. Therefore recent “guidelines” [1] have suggested that, in the absence of target organ damage or previous events there is no clear justification to initiate pharmacologic treatment in subjects with WCH although periodic monitoring (including a ABPM/year) and surveillance is required. Thus ABPM may be considered an important technique for evaluation of new hypertensive patients even in the primary care setting. There are virtually no studies totally performed in primary care that have so far evaluated the diagnostic accuracy of the ABPM and its discriminative value of cardiovascular risk. Thus based on the identification of WCH with ABPM, the aim of this study was to assess the diagnostic accuracy and to determine the cost-benefit ratio of 24-hour ambulatory blood pressure monitoring (ABPM) in a population of newly diagnosed and previously untreated hypertensive patients (HTs) attended in primary care.

## Methods

We conducted a cross-sectional and analytical observational study consisting on the analysis of the ABPM results and other clinical variables, taken from all 360 newly hypertensives patients diagnosed from July 2006 to November 2007 by their GP. All patients were fully observed in the Health Center of São João in Oporto and ABPM evaluation was performed in all patients before any treatment was prescribed. All study was carried out in compliance with the Helsinki Declaration, and its protocol was approved by the Ethical Committee of Centro Saude S. Joao, Porto, Faculdade Medicina do Porto 4/2006 and all patients participated the study under their written informed consent. The following variables were recorded: office BP, data from ABPM, gender, age, BMI, smoking habits, dyslipidemia, diabetes mellitus and family history of cardiovascular disease. Office BP values (phases I and V of Korotkoff) were redorded in the left arm with Omron M6 sphygmomanometer (average of 3 recordings with 5 min apart), using an appropriate bladder, after resting for 10 minutes in a sitting position and repeated 8–15 days thereafter. For ABPM data were used a SpaceLabs 90207 device (SpaceLabs Inc, Redmond, Washington, USA), connected to the non-dominant arm. Blood pressure was recorded during a normal working day with intervals of 20/20 minutes during the daytime (07:00 to 23:00) and of 30/30 minutes during the nighttime resting period (23:30 to 06:30). The nocturnal fall of BP was determined by the (average daytime BP - average nighttime BP)/daytime average BP × 100. Patients were classified as dippers for nocturnal systolic BP decline between 10–19,9%, non - dippers for nocturnal BP decline between 0–9,9%, inverted-dippers for nocturnal BP decline between <0% and the extreme dipper for nocturnal BP fall ≥ 20%. Normotension was defined if casual BP was <140 mm Hg (SBP) and <90 mm Hg (DBP) and values of the average daytime ambulatory BP <135 and <85 mm Hg. Hypertension was defined for casual BP values ≥ 140 and/or 90 mm Hg and average daytime ambulatory BP ≥ 135 and/or 85 mm Hg. WCH was defined for casual BP values ≥ 140 and/or 90 mm Hg and values of average daytime ambulatory BP <135 and <85 mm Hg. Concerning the presence of cardiovascular risk factors we established an index of overall risk per patient resulting from the arithmetic sum of each one (presence = 1, absence = 0) of the following risk factors: ageing > 65 years in women and > 55 years in women, obesity (BMI ≥ 30 kg/m2), dyslipidemia i.e. total cholesterol > 190 mg/dl) and/or HDL-C <46 mg/dl women and <40 mg/dl men, and/or triglycerides > 150 mg/dl, smoking and family (first and second degree relatives) history of cardiovascular events before 65 years.

We used a model to calculate cost-effectiveness of ABPM in primary care taking into account the cost of medical assistance (drug treatment, medical visits and laboratorial evaluation) of hypertensive patients diagnosed in office vs the cost of medical assistance with a strategy based on use of ABPM hypertensive patients, allowing to select true hypertensives and subjects with WCH.

The model was estimated for a total of 1000 patients diagnosed as hypertensives according to BP measured in office and followed-up for 2 years with and without information of ABPM data. The following parameters were considered: a) Prevalence of WCH in the initial evaluation by ABPM; b) cost of ABPM (65 euros/registration), c) cost of treatment antihypertensive (0.65 euros/day, when indicated, d) cost of routine analytical evaluation in hypertensive patients (14.42 euros for each assessment) and of the minimum number of medical visits (cost 41 euros/visits, e) the cost of medical visits considered minimally required i.e. two medical visits and two analytical evaluations per year for all hypertensives diagnosed in office without ABPM evaluation and for all true hypertensive patients confirmed by ABPM versus a single visit and a single analytical evaluation per year for individuals with WCH), e) gain (savings) estimated in euros for the period of 2 years using ABPM (avoiding pharmacological treatment in individuals with WCH and half reducing the number of medical visits and screening summary) minus the cost of ABPM versus the cost of medical assistance in all patients diagnosed as hypertensive in the absence of use of ABPM. The estimation of cost of drug therapy was based on “Medicinal products for human use for use in outpatient clinic, INFARMED, Portugal 2006” and the cost of medical ambulatory visits and of diagnostic procedures was based on Portuguese National Health System 2006 price list of diagnostic techniques [26].

### Statistical analysis

The SPSS (Statistical Package for Social Science) version 12.0 was used to perform statistical analysis. Parametric data are as average of ± SD. We used the chi-square to compare groups for categorical variables. The differences between the group of sustained hypertensives and the group of subjects with white-coat hypertension events were obtained by statistical parameter using the *t* test Student for independent groups, analyzing the following variables: age, sex, biochemical variables, casual BP, 24-hour, daytime and nightime records of ABPM. All tests were considered statistically significant for significance level (p) <0.05.

## Results

Office BP and ABPM evaluations were performed in 360 patients with newly hypertension diagnosed in office, without antihypertensive medications who were attended in General Practice/Family Medicine between July 2006 and November 2007 (16 months). Out of these, 24 individuals were excluded since casual BP in the second recording day evolve towards normotensive values. Therefore for this study we included 336 individuals, 56% women, ageing 51 ± 14 (15–82) years, BMI 26.5 ± 4 (17–41) kg/m2; 3.3% were diabetics, 20.8% had BMI > 29.9 kg/m2, 20.2% were smokers, 44.6% had dyslipidemia, 3% had previous strokes and 1% previous coronary even and 15.5% had a family history of hypertension. After ABPM we identified 206 as true hypertensive patients (HTs, 61.3%) and 130 as having white coat hypertension (WCH, 38.7%). Table 1 summarizes and compares the clinical characteristics of these two subgroups. Compared to WCH, the HTs showed significantly higher levels of casual BP, mean 24 h, daytime and nighttime ambulatory pressures, blood pressure on awakening and 24-hour heart rate. The group of HTs also showed significantly higher blood levels of triglycerides, and of the composite index made by the association of various cardiovascular risk factors. Also, the percentage of males and smokers was significantly greater in HTs vs WCH groups and there were not statistically significant differences concerning the percentage of obese, of diabetics and of patients with dyslipidemia. As shown, there were no statistically significant differences between HTs and WCH regarding age, BMI, blood levels of fasting glucose and total cholesterol and HDL-C sub fraction. Also there were no differences between groups for different patterns of nocturnal decline in BP. Table 2 shows the calculation of cost of medical resources of either strategies with and without the contribution of ABPM, estimated for 1000 hypertensive patients classified with office BP followed for two years. Based on data from the present study we considered that after ABPM, WCH would be diagnosed in 38% of the hypertensive patients identified only with office BP. As shown, on the strategy without ABPM and according to guidelines [27] all hypertensive patients diagnosed in office as well as all true hypertensive patients diagnosed with ABPM should be treated with antihypertensive drugs and submitted per year at least to two doctor visits and to two routine biochemical evaluations. In contrast follow-up of subjects with WCH (i.e. 38% of all hypertensive patients diagnosed in office) could be restrained per year to a single doctor visit and to a single routine biochemical evaluation whereas they also should perform an ABPM recording every year. Despite of ABPM strategy involving the additional cost of this examination we can obtain an overall saving of about 23% of resources i.e. corresponding to around 157,500 euros for 1000 patients followed for two years in general practice.

**Table 1:** Anthropometric characteristics and values of casual blood pressure, ambulatory BP and of biochemical data in individuals classified as WCH and HTs

	**WCH (n = 130)**	**HTs (n = 206)**	***p***
**Age (years)**	50 ± 14	52 ± 14	0.27
**Women (%)**	68	51	<0.05
**BMI (kg/m**^**2**^**)**	26 ± 4	27 ± 4	0.35
**BMI >29.9 Kg/m2 (%)**	18	23	0.32
**Smokers (%)**	14	24	<0,01
**Dyslipidemia (%)**	38	47	<0.05
**Diabetes (%)**	2.3	3.9	0.19
**Family history of CV disease (%)**	13	20	0.23
**Office SBP (mm Hg)**	154 ± 12	161 ± 15	<0,001
**Office DBP (mm Hg)**	92 ± 10	94 ± 9	<0,05
**24 h SBP (mm Hg)**	120 ± 6	135 ± 10	<0,001
**24 h DBP (mm Hg)**	73 ± 5	85 ± 7	<0,001
**24 h PP (mm Hg)**	47 ± 6	51 ± 9	<0,001
**24 h Heart rate (beats/min)**	73 ± 9	77 ± 9	<0,001
**Daytime SBP (mm Hg)**	124 ± 6	140 ± 10	<0,001
**Daytime DBP day (mm Hg)**	77 ± 5	89 ± 8	0.052
**Nighttime SBP (mm Hg)**	113 ± 8	126 ± 12	<0,001
**Nighttime DBP day (mm Hg)**	66 ± 6	76 ± 8	<0,001
**Nocturnal SBP fall (%)**	8,7 ± 5,6	10,1 ± 5,6	<0,05
**Inverted-dipper (%)**	2	4	0.44
**Non-dipper (%)**	43	46	0.62
**Dipper (%)**	46	47	0.71
**Extreme-dipper (%)**	9	3	0.11
**SBP on awakening (mm Hg)**	116 ± 15	134 ± 17	<0,001
**Total Cholesterol (mg/dl)**	216 ± 37	220 ± 39	0.12
**HDL-C (mg/dl)**	58 ± 15	54 ± 17	0.14
**Tryglicerides (mg/dl)**	128 ± 83	160 ± 124	<0,05
**Glicemia (mg/dl)**	95 ± 19	101 ± 26	0.09
**numRF (n)**	1,15 ± 0,90	1,44 ± 0,98	<0,05

Values expressed as average ± standard deviation.

*WCH* White Coat Hypertensive, *HTS* Sustained Hypertensive, *BMI* Body Mass Index, *SBP* Systolic blood pressure, *DBP* Diastolic blood pressure, *HR* heart rate; *PP* Pulse Pressure, *numRF* Number of risk factors, *p* level of significance.

**Table 2:** Reducing costs on medical resources with the use of ABPM in 1000 hypertensive patients followed at the doctor’s office for 2 years, allowing the identification of WCH (prevalence of 38%)

**1000 patients followed for 2 years**	**Prevalence of HT to treat**	**Cost of treatment in Euros (0,65 Euros/day)**	**Doctor visits cost (41 Euros/visit)**	**Biochemical analysis cost (14,42 euros/analysis)**	**ABPM cost 65 Euros/ABPM**	**Total cost (euros/2 years)**	**ABPM saving strategy (euros/2 years)**
Without ABPM	100%	474500	164000	57680	0	696180	0
With ABPM	62%	294190	132840	46721	65000	538751	157429
% ABPM saving strategy							22,6%

## Discussion

The aim of this study was to evaluate in Primary Care, the diagnostic value and cost-effectiveness of ABPM in Primary Care in newly diagnosed hypertensive patients. Because benefits from drug treatment in white-coat hypertension (WCH) remain unproven, we estimated that the avoidance of drug treatment and the reduction of other medical care expenses in the 38% of the hypertensive patients diagnosed as having WCT would represent an overall significant saving of medical resources and cost of therapy if 24-hour ABPM was generalized to all hypertensive patients diagnosed only on the basis of casual BP measurements.

The importance of ABPM in the diagnosis, monitoring and prognostic stratification of hypertensive patients is clearly defined in literature [1,7-9,11-14,22,28-32]. ABPM is considered a simple and noninvasive technique with a higher diagnostic accuracy comparing to office BP providing exclusive data on the blood pressure profile in real life outside the medical office [1,7-9,11-14,22,28-32]. ABPM is a valuable tool for the diagnosis of masked hypertension a condition cursing with normotensive values in office and abnormally high pressure values outside the office setting that has been associated with an increased incidence target organ damage [33]. ABPM also allows to identify those individuals with white coat hypertension/isolated office hypertension (WCH) [1,4,6,12,16,21,24,34],[35] who exhibit a high blood pressure values in the doctor’s office in contrast with normal blood pressure values outside that setting. WCH cannot be diagnosed reliably on clinical examination alone. Both ABPM and home blood pressure monitoring (HBPM) overcomes many of the limitations of traditional office blood pressure (BP) measurement and are the mostly used tools for the diagnosis of white-coat hypertension [1]. Home readings are more reproducible than office readings and show better correlations with measures of target organ damage [12]. Since it is less influenced by the white-coat effect HBPM contributes for the diagnosis of WCH. However, using ABPM as a reference, it has been shown a lower accuracy, specificity and sensitivity of HBPM to detect white coat hypertension [36] and that HBPM may not be completely devoid of white-coat effects [37]. Although there are some potential cost-effectiveness advantages for the use of HBPM in the diagnosis and management of hypertension by reducing the need for office visits, the net cost of home blood pressure monitorings and the absence of reimbursement by National Health Systems, as it occurs in Portugal, limits it generalization to a broad population and threatens its potential cost-effectiveness [38]. WCH has been associated with a low cardiovascular risk profile at least while comparing to sustained hypertensives [1,4,8,11,14,23-25,39]. Careful surveillance and implementation of healthy lifestyles but not pharmacological therapy has been recommended in WCH [1,4,24,25] and no study has so far clearly proven that drug treatment provides any benefit. Thus, the identification of WCH may have important consequences in reducing medical expenses related with drug treatment of hypertension, reducing the need of medical visits and exams [40]. Almost all studies using 24 hours ABPM have been made so far in hospitals. i.e. outside the Primary Health Care Centers. Since hypertension is highly prevalent in general adult population (about 40% of adult population) most patients are diagnosed, evaluated and followed in general practice. In our study, for the estimation of the diagnostic value and cost-effectiveness of ABPM in Primary Care we evaluated clinically and with ABPM 336 adult patients with newly diagnosed office hypertension, that in 38.7% were fulfilling the diagnosis of white coat hypertension. This percentage of WCH as well as a higher number of women in this group is consistent with that found in the literature [3,5,12,13] particularly concerning the hypertensive population with stage 1 and 2 hypertension at the office, i.e. the population that is more frequently observed in Primary Health Care. For a similar age and BMI distribution, the group of individuals with sustained hypertension shoed higher casual blood pressure and 24-hour ABPM values comparing to WCH. True hypertensives also showed a worse profile of global cardiovascular risk as expressed in the coexistence of a cumulative aggregate of cardiovascular risk factors, beyond the office BP levels. Also, office BP and ABPM values were higher in HTs individuals than in those with WCH. Thus, the present study confirms that patients with WCH show several indicators of a lower cardiovascular risk than patients with sustained hypertension. There is now consensus that individuals with WCH require medical supervision but benefit from pharmacological treatment is unproven [1,24,25,29]. Thus the diagnosis of this condition could have important implications in reducing health expenses on medication, medical visits and diagnostic exams that could result in the reduction of iatrogenic risks and of direct and indirect (personal, family and social, etc.) costs.

In the present study we estimated the cost benefit of two strategies of monitoring of newly diagnosed hypertensive patients based and not based on the general use of 24-h BPM in Primary Health Care. With ABPM we were able to identify 38% of the hypertensive patients diagnosed in office as having WCT. Although ABPM strategy implied an increased cost related with generalized 24-h BP monitoring procedure, there was a significant saving of medical resources and cost of therapy since these subjects were not only restrained from drug treatment but also doctor visits and routine biochemical evaluation were reduced by half as compared with sustained hypertensives.

Considering the high prevalence of the WCH found in the newly diagnosed hypertensive population, our study clearly suggests that the implementation of a strategy of widespread use of ABPM in Primary Care provides a highly favourable cost-benefit ratio. Our estimation for 1000 patients followed for 2 years showed that it is possible to reduce about 23% of total medical expenses with a strategy based on ABPM vs no use of ABPM. In other words ABPM based strategy reduced overall expenses by 157.500 euros per 1000 patients evaluated for two years. These data agree to those of other studies in which ABPM showed a favourable benefit-cost ratio for optimizing therapy [24,40] and for reducing the number of antihypertensive drugs needed in patients with sustained hypertension under chronic treatment [41]. Meanwhile, our estimation did not take into account any additional gain resulting from the avoidance of possible drug side effects and iatrogenic related costs associated with the massive and widespread use of medication in subjects for whom the benefits of therapy have not been proved. Our study has limitations. Beyond the factor related with the relatively small dimension of our sample it is generally assumed that 24-h ABPM is not easily available to all Private care Doctors thereby limiting the generalization of this method. Secondly it is not clear whether subjects with WCH who has concomitant other risk factors beyond office hypertension or underlying diseases do not require the immediate start of anti-hypertensive treatment. Also in our study there was another expected inconvenience related to a possible misdiagnosis of hypertension at entry while based only in casual BP evaluation that could lead to the inclusion in the WCH group some real normotensive subjects.

## Conclusions

In conclusion our study shows that in Primary Health Care, the strategy based on the widespread use of 24-hour ABPM in newly diagnosed hypertensive patients has a highly favourable benefit-cost ratio in comparison with a strategy without ABPM. The former strategy allows to reduce by 23% the overall costs associated with the implementation of therapy and of medical diagnosis and monitoring procedures. Our data may support the widespread implementation of the ABPM technique in general medicine/family practice within the approach of newly hypertensive patients, by contributing to increase diagnostic accuracy of hypertension, to improve risk stratification and to reduce the costs and the iatrogenic drug risks.

## Competing interests

All authors declare that they have no financial or non-financial competing interests concerning the present study.

## Authors’ contributions

PP was the doctor responsible for local supervision of the collection of clinical data and also attended 40% of the included subjects. MV and PF were the clinicians who attended more than 60% of the patients thereby performing the collection of clinical data. SB was responsible for collection of ABPM data and to include it in a data base. JP conceived the study and its design, wrote the manuscript, elaborate the answers to the reviewers and performed the statistical analysis. All authors equally participate in the general design of the study and in the discussion of the results. All authors read and approved the final manuscript.

## Pre-publication history

The pre-publication history for this paper can be accessed here:

http://www.biomedcentral.com/1471-2261/13/57/prepub
